# SDN TCP-SYN Dataset: A dataset for TCP-SYN flood DDoS attack detection in software-defined networks

**DOI:** 10.1016/j.dib.2025.111314

**Published:** 2025-01-16

**Authors:** Sudesh Kumar, Sunanda Gupta

**Affiliations:** School of Computer Science and Engineering, Shri Mata Vaishno Devi University, Katra, India

**Keywords:** Software Defined Networking, Artificial Intelligence, Network security, Anomaly detection

## Abstract

The TCP-SYNC SDN dataset, a collection of labelled network traffic data for investigating DDoS attack detection in Software-Defined Networking (SDN) environments. The dataset includes flow-level metrics from both normal and malicious traffic, particularly TCP-SYN flood attacks. Generated in a controlled testbed using advanced simulation tools Mininet(v2.3.0) and real internet traffic captured from real-world internet browsing sessions via Google Chrome and recorded using Wireshark(v3.0.2), this dataset is essential for researchers and practitioners working on TCP-SYN flood DDoS attack detection and machine learning-based traffic classification. The dataset is publicly available at Mendeley Data.

Specifications Table

**Table:** 

Subject	Cryptography and Cybersecurity/Artificial Intelligence
Specific subject area	Software-Defined Networking (SDN), DDoS Detection
Type of data	Tabular,CSV
Data collection	The dataset was generated in a controlled SDN testbed environment using Mininet(v2.3.0), a network emulation tool. Normal traffic was captured from real-world internet browsing sessions via Google Chrome and recorded using Wireshark(v3.0.2). Hping3(v3.0.0-alpha-2) tool was used to simulate TCP SYN flood DDoS attack in SDN.
Data source location	Institution: Shri Mata Vaishno Devi University City/Town/Region: Katra/Jammu & Kashmir Country: India Latitude: 32.940391° Longitude: 74.954906°
Data accessibility	Repository name: Mendeley Data Data identification number: 10.17632/236bd4cjmk.2 Direct URL to data: https://data.mendeley.com/datasets/236bd4cjmk/2
Related research article	‘none’

## 1 Value of the Data

• **TCP-SYN Flood DDoS Attack Detection:** The Data is useful for Machine Learning Model Development for TCP-SYN Flood DDoS attack detection in software defined networks, as of now no TCP-SYN flood SDN DDoS dataset is available with realistic internet traffic [1].
• **Realistic Simulation:** Dataset Includes both real-world internet traffic captured from real-world internet browsing sessions via Google Chrome subsequently recorded using Wireshark (v3.0.2) and simulated TCP-SYN flood DDoS attacks [2].
• **Reproducibility:** Detailed experimental setup and pre-processing ensure reproducibility for academic and industry use.
• **Feature-Rich Dataset:** Dataset contains 84 detailed flow-level features useful for in-depth network traffic analysis.

## 2 Background

The original motivation behind compiling the TCP-SYN SDN dataset stems from the need to address the challenges of detecting Distributed Denial of Service (DDoS) attacks in Software-Defined Networking (SDN) environments. DDoS attacks, particularly TCP-SYN flood attacks, are a significant threat to network security due to their ability to overwhelm network resources. Despite extensive research in this area, there is a lack of publicly available datasets specifically focused on TCP-SYN flood attacks in SDN environments, which limits the development and evaluation of robust detection mechanisms.The dataset was generated using a controlled SDN testbed, leveraging Mininet for network emulation, Wireshark for traffic capturing, and Hping3 for simulating TCP-SYN flood attacks. By incorporating both real-world internet traffic and simulated malicious traffic, the dataset provides a realistic foundation for studying traffic patterns and developing machine learning models for DDoS detection. The design of the dataset ensures a comprehensive representation of network flows, capturing 84 detailed features for in-depth analysis. This initiative aims to support researchers and practitioners by providing a reproducible, feature-rich dataset that facilitates advancements in anomaly detection and network security within SDN frameworks.

## 3 Data Description

The provided dataset contains flow-based network traffic data captured in an SDN environment during TCP-SYN flood attack experiments. The dataset includes **6,233 entries** with **84 features** per entry, encompassing details about the network flows, packet-level statistics, and temporal properties [3]. Below is a detailed description of the dataset:

**1.1. Structure of the Dataset**

• **Number of Entries:** 6,233
• **Number of Features:** 84
• **Data Types:**
  ∘ **Object (Categorical):** 5 columns
  ∘ **Integer (Numerical):** 42 columns
  ∘ **Float (Numerical):** 37 columns

**2.1. Key Features**

• **Flow Metadata:**
  ∘ Flow ID: Unique identifier for each flow.
  ∘ Src IP, Dst IP: Source and destination IP addresses.
  ∘ Src Port, Dst Port: Source and destination port numbers.
  ∘ Protocol: Protocol used in the flow (e.g., 6 for TCP).
  ∘ Timestamp: Timestamp for the flow start.
• **Traffic Statistics:**
  ∘ Flow Duration: Duration of the flow in microseconds.
  ∘ Tot Fwd Pkts, Tot Bwd Pkts: Total forward and backward packets in the flow.
  ∘ TotLen Fwd Pkts, TotLen Bwd Pkts: Total length of forward and backward packets.
• **Packet Length Metrics:**
  ∘ Fwd Pkt Len Min, Fwd Pkt Len Max, Fwd Pkt Len Mean, Fwd Pkt Len Std: Metrics for forward packet lengths between source and destination.
  ∘ Bwd Pkt Len Min, Bwd Pkt Len Max, Bwd Pkt Len Mean, Bwd Pkt Len Std: Metrics for backward packet lengths between source and destination.
• **Flow Rate Metrics:**
  ∘ Flow Byts, Flow Pkts: Bytes per second and packets per second for the flow.
  ∘ Fwd Pkts, Bwd Pkts: Forward and backward packets per second.
• **Inter-Arrival Time (IAT):**
  ∘ Flow IAT Mean, Flow IAT Std, Flow IAT Max, Flow IAT Min: Mean, standard deviation, maximum, and minimum of inter-arrival times within flows.
  ∘ Fwd IAT Mean, Bwd IAT Mean: Inter-arrival times for forward and backward packets.
• **Flags and Header Statistics:**
  ∘ SYN Flag Cnt, ACK Flag Cnt, FIN Flag Cnt: Counts for TCP flag occurrences in the flow.
  ∘ Fwd Header Len, Bwd Header Len: Forward and backward header lengths.
• **Behavioral Metrics:**
  ∘ Active Mean, Idle Mean: Mean active and idle durations of the flows.
  ∘ Down/Up Ratio: Ratio of downstream to upstream traffic.

**3.1. Labels**

• **Target Variable:** Label
  ∘ Binary labels indicating the type of traffic:
    • **Normal**: Legitimate traffic flows.
    • **Attack**: TCP-SYN flood attack flows.
  ∘ This labelling is essential for supervised machine learning-based DDoS attack detection.

This dataset is well-suited for training and evaluation of machine learning models for DDoS attack detection in SDN environments [4]. The comprehensive features and balanced labelling provide detailed insights into traffic patterns, facilitating accurate classification of network flows.

## 4 Experimental Design, Materials and Methods

To analyse and detect TCP-SYN flood Distributed Denial of Service (DDoS) attack in Software-Defined Networking (SDN), it is essential to generate realistic datasets that capture both normal and attack traffic. The proposed methodology describes the systematic implementation of a TCP-SYN flood attack dataset in an SDN environment using the Mininet emulator, controlled by a RYU SDN controller. The TCP-SYN flood, a volumetric attack, aims to exhaust the network's resources by overwhelming the server with a high volume of SYN requests, simulating real-world attack scenarios.

**1. Testbed Specification** followings are the list of hardware's and software's required for simulations environments:

• **Hardware:** Intel(R) Core(TM) i7-7500U CPU @ 3.0GHz, 16GB RAM.
• **Operating System:** Ubuntu 20.04.2 LTS.
• **Network Simulation Tools:** Mininet (v2.3.0) and Hping3 (v3.0.0-alpha-2).
• **Traffic Generation Tools:** Captured real-world internet traffic using Google Chrome(v80.0) and Wireshark(v3.0.2); Hping3 for attack traffic.

**2. Network Topology Design**

**Fig. 1:**
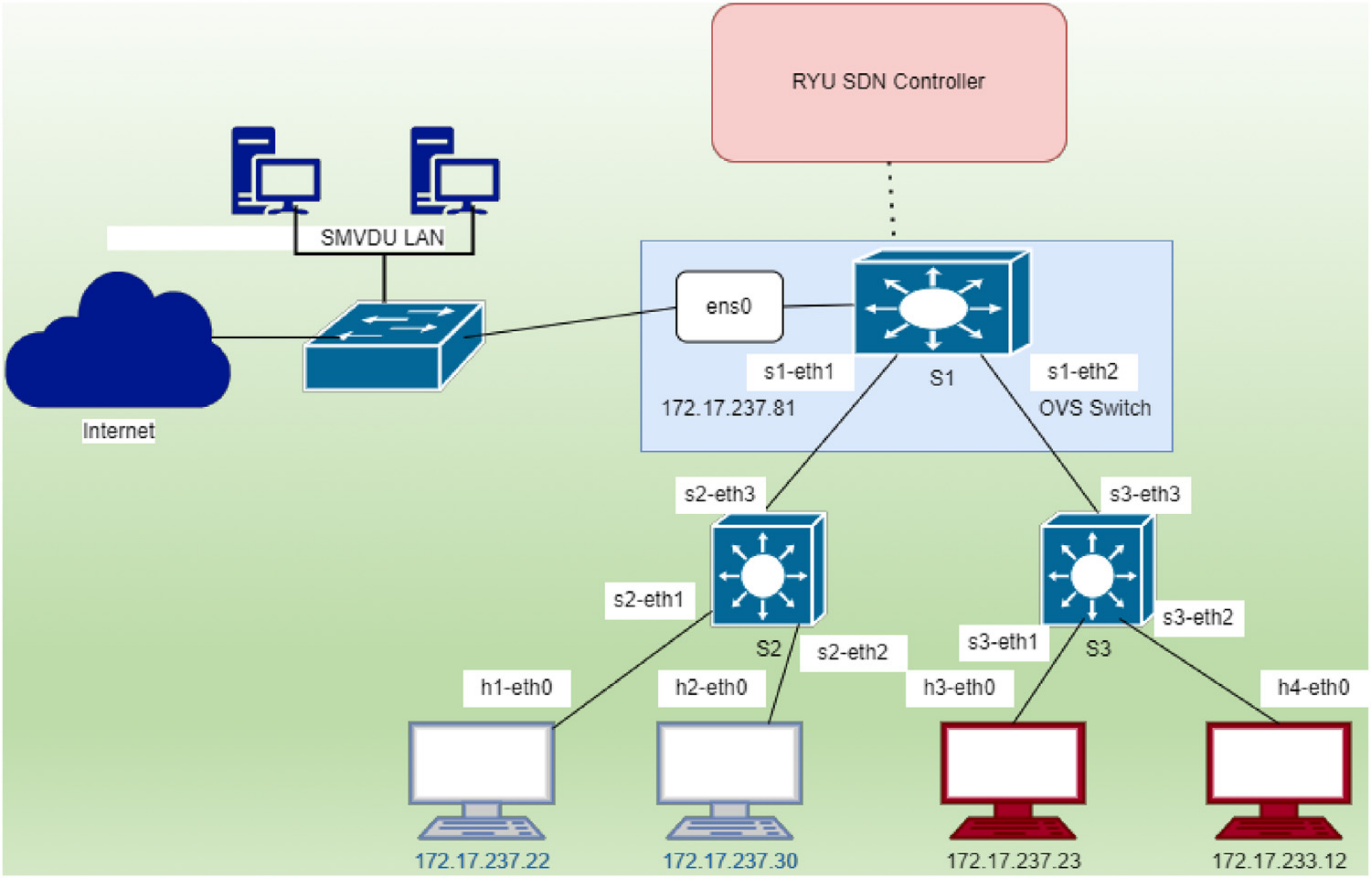
Network topology with RYU controller.

The network topology as depicted in Fig. 1, was designed and implemented using Mininet, an SDN network emulator, to create a virtual environment for generating a TCP-SYN flood dataset. The topology consists of a central Open vSwitch (S1) connected to two subordinate switches (S2 and S3) and a set of end hosts. The network is managed by the RYU SDN controller, which dynamically programs the flow tables of the switches using the Open Flow v1.3 protocol. Hosts h1 and h2 simulate legitimate traffic, while hosts h3 and h4 act as attack generators, producing malicious TCP-SYN flood traffic. The entire setup connects to the SMVDU LAN and the Internet through the ens0 interface of S1, enabling real-world traffic simulation. This design ensures the topology can support diverse traffic patterns, including normal and attack scenarios.

**3. Experimental Setup in Mininet**

**Fig. 2:**
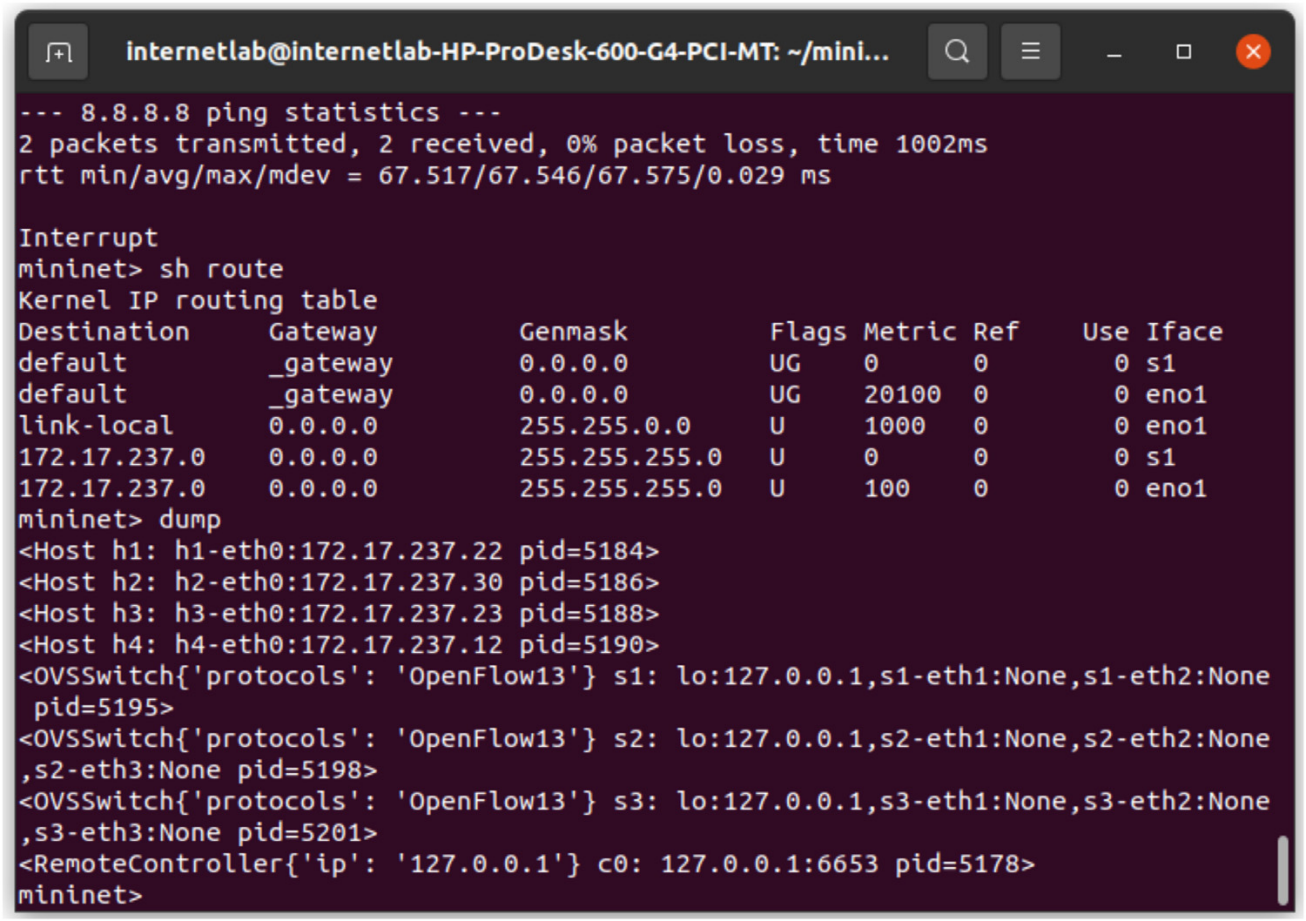
hosts configuration setup on Mininet emulator.

The SDN environment was emulated in Mininet, where switches, hosts, and links were configured as per the defined topology as demonstrated in Fig. 2. The connectivity between all hosts was verified using the pingall command, ensuring the network setup was functional. The RYU SDN controller, deployed as a remote controller, managed traffic flows by monitoring packet transmissions and dynamically installing flow rules to handle network behaviour under both normal and attack conditions. Legitimate traffic was generated by browsing various categories of websites from hosts h1 and h2 using google chrome browsers in SDN environments, while malicious traffic was simulated using h3 and h4 to create a high volume of SYN packets targeting legitimate hosts.

**4. Implementation Details**

**Fig. 3:**
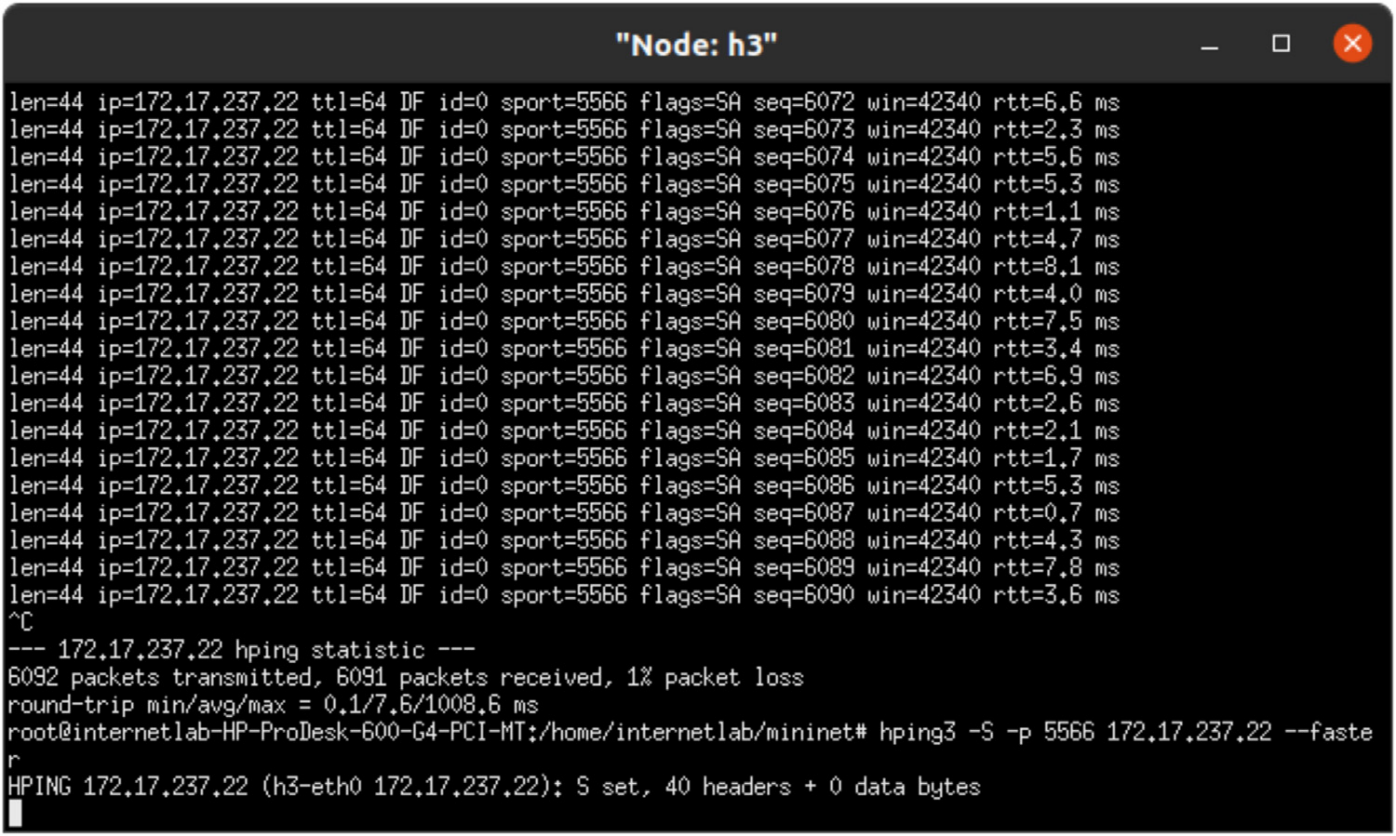
Node h3 flooding packets to node h1.

The Mininet CLI was utilized to initialize the network with the command mn -–controller= remote, ip=127.0.0.1 –topo=tree,2,2 –mac –switch ovs, protocols=OpenFlow13. As shown in Fig. 3, TCP-SYN flood attacks were generated using the hping3 tool, where malicious hosts (h3 and h4) flooded the network with SYN packets targeting h1 and h2 using commands like hping3 -S -p 5566 172.17.237.22 –faster. Simultaneously, Wireshark was deployed to capture packet-level data and detailed traffic analysis.

**5. Resource Utilization Statistics**

**Fig. 4:**
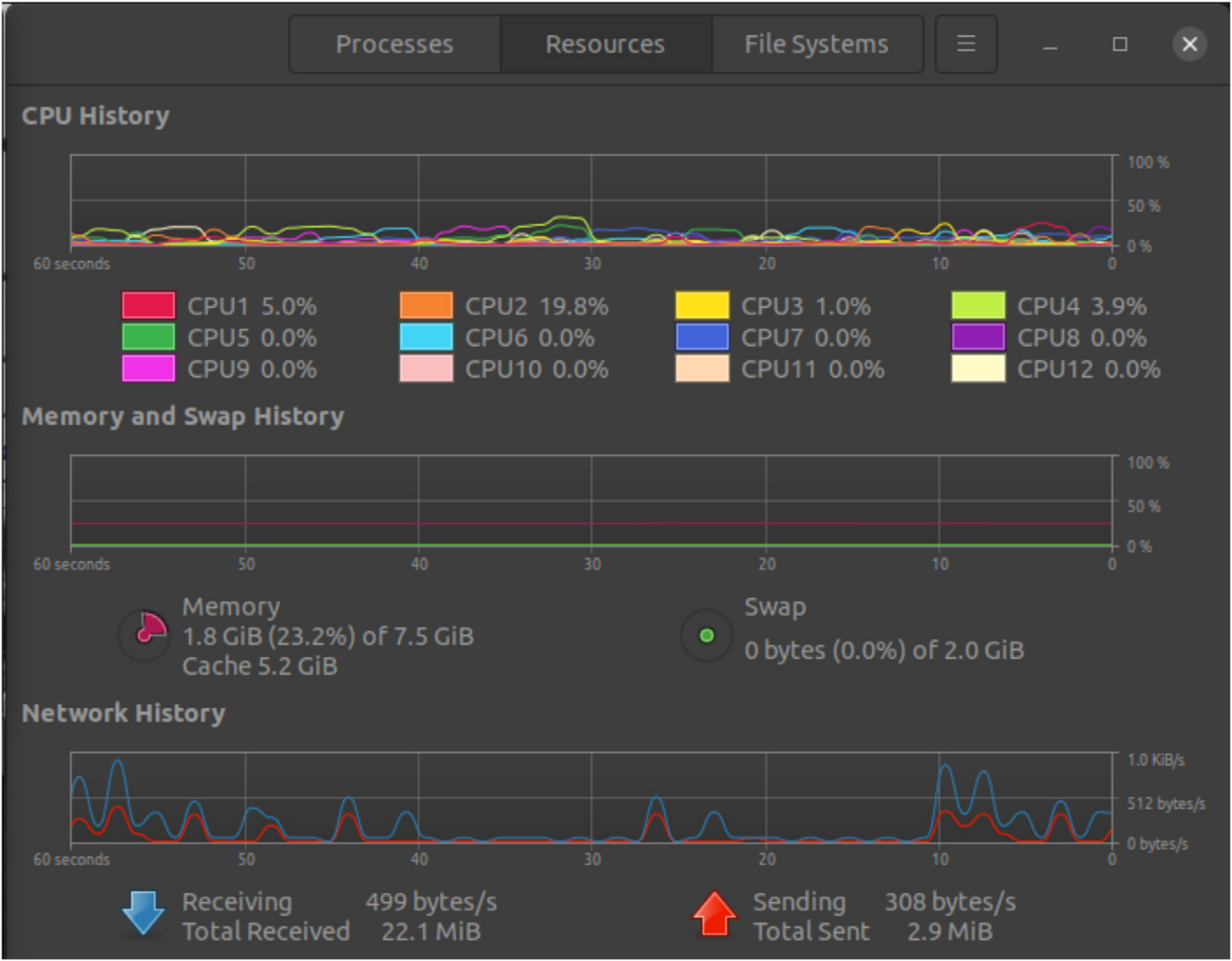
Resources utilization under normal traffic.

**Fig. 5:**
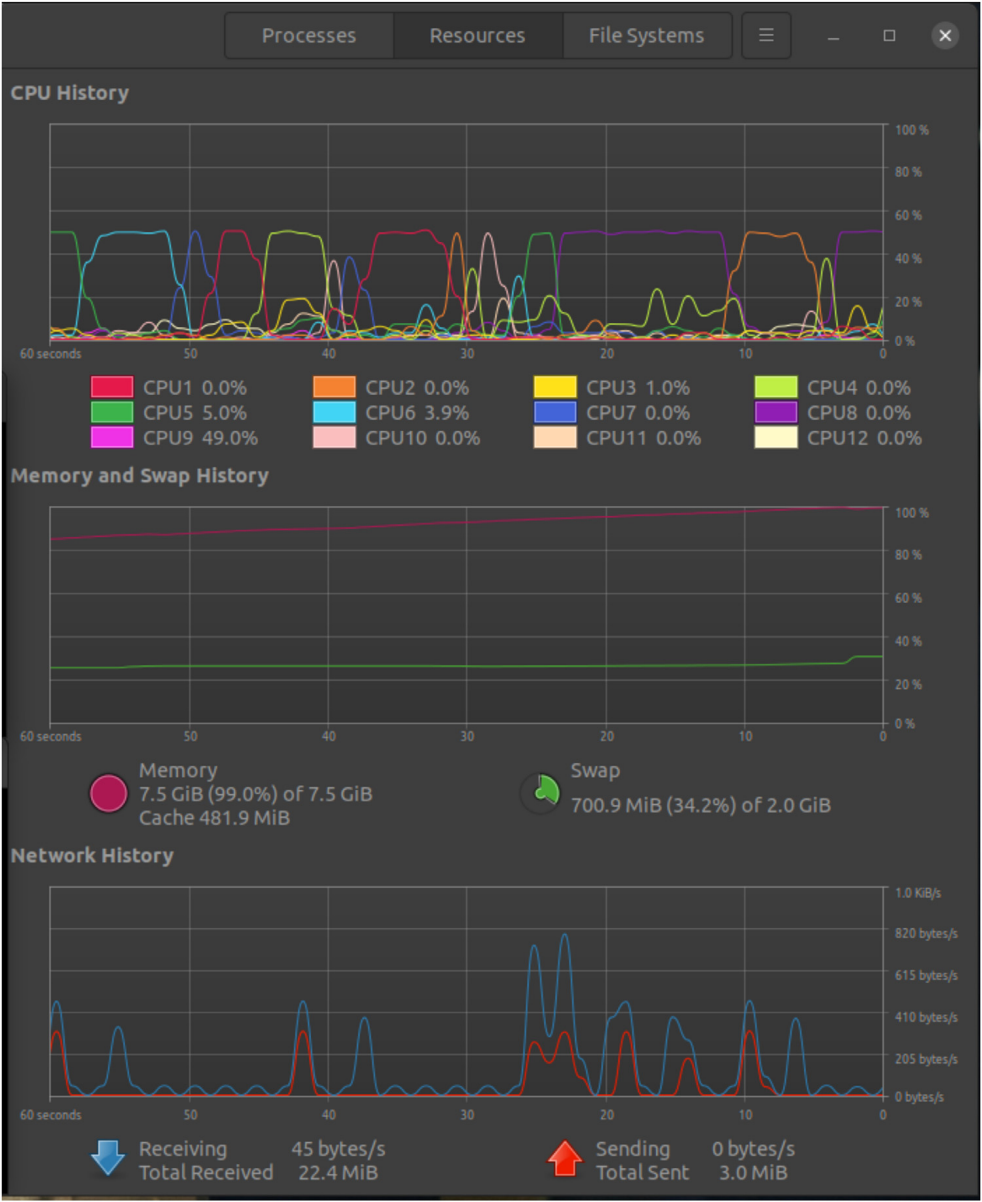
Resources utilization under attack traffic.

Resource utilization during the experiment was closely monitored to understand the impact of DDoS attacks on the SDN environment. Fig. 4 shows resources utilization under normal traffic. CPU and memory usage spikes were observed on the switches and the RYU controller, reflecting the increased overhead required to process high volumes of SYN packets. Bandwidth utilization also surged significantly, with sharp peaks during attack traffic generation. System resource utilization data, including CPU, memory, and network bandwidth, was visualized using resource graphs Fig. 5, highlighting the performance strain caused by malicious traffic. These statistics provided a detailed understanding of the resource consumption and system behaviour under varying traffic conditions, emphasizing the need for efficient detection and mitigation strategies in SDN environments.

**6. Dataset Collection and Traffic Capturing Using Wireshark**

**Fig. 6:**
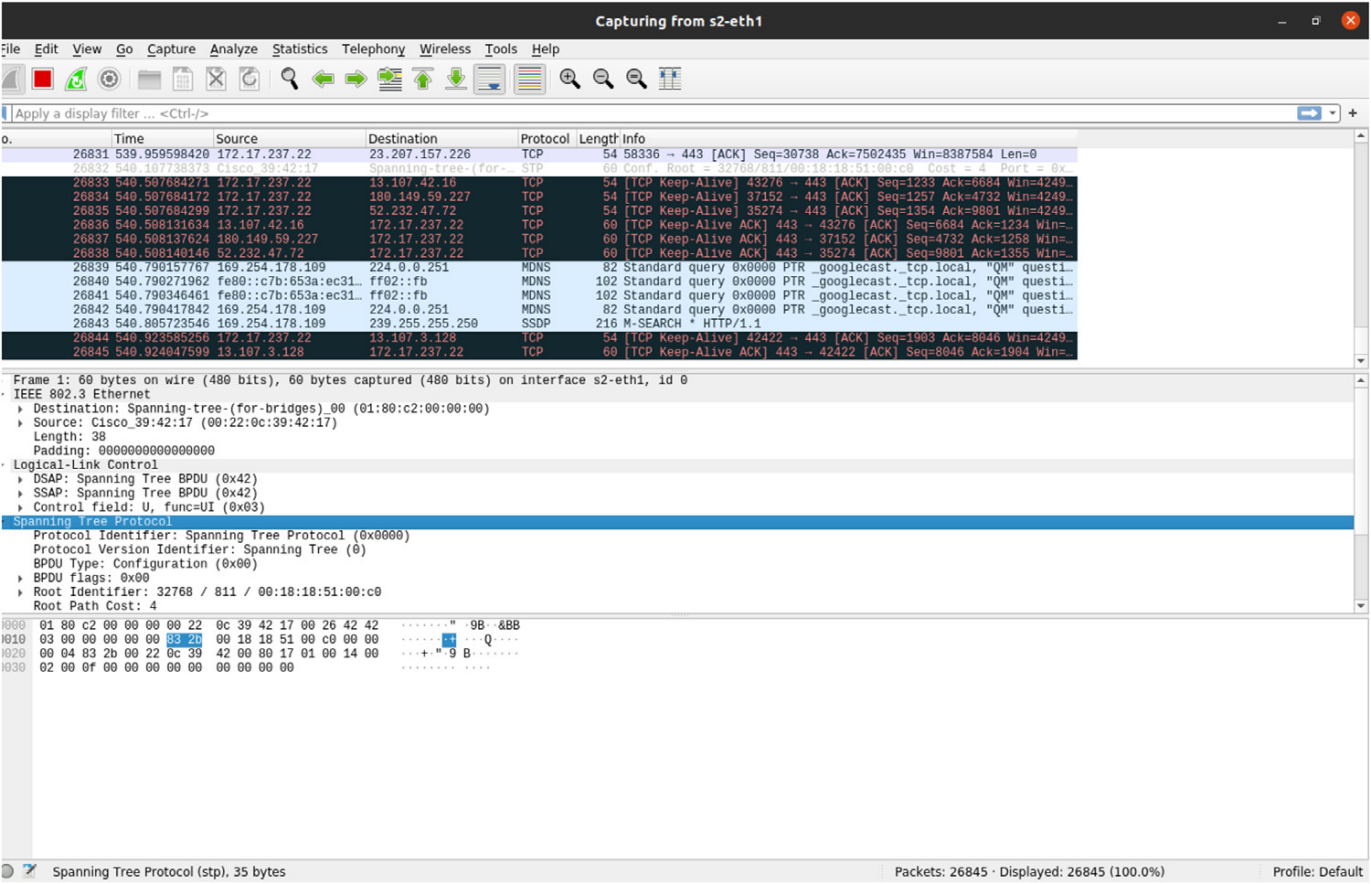
Traffic capturing using Wireshark.

The dataset was generated by capturing and labelling traffic flows from the experiment. As demonstrated in Fig. 6, Wireshark was used to monitor and capture traffic, recording key packet details such as source and destination IPs, TCP headers, packet lengths, and flow durations. The collected data provided insights into traffic patterns, distinguishing between normal bidirectional flows and one-sided malicious SYN packet floods. Captured traffic was labelled as “Normal” or “Attack” based on the behaviour and source, where legitimate traffic was labelled as 0, and malicious traffic was labelled as 1. This labelled dataset was structured for training and evaluating machine learning-based DDoS detection mechanisms.

## Limitations

“None”

## Ethics Statement

The authors confirm adherence to the ethical standards required for publication in *Data in Brief*. This study did not involve human subjects, animal experimentation, or the use of data from social media platforms.

## Credit Author Statement

**Sudesh Kumar:** Conceptualization, Investigation, Methodology, writing – original draft preparation; **Dr. Sunanda Gupta:** Conceptualization, Methodology, Writing – review & editing, Supervision.

## Data Availability

Mendeley DataSDN-TCP-SYN ATTACK-DDOS DATASET (Original data).
